# Implementation of a Community-Based Psychosocial Support Focal Point Response for Internally Displaced Persons in Myanmar During COVID-19

**DOI:** 10.3389/fpubh.2022.854490

**Published:** 2022-03-24

**Authors:** Catherine Lee, Matthew Schojan, Ko Myo, Gyaw Htet Doe, Lanau Htu San, Judith Bass

**Affiliations:** ^1^Bloomberg School of Public Health, Johns Hopkins University, Baltimore, MD, United States; ^2^Substance Abuse Research Association, Yangon, Myanmar; ^3^Kachin Baptist Convention, Myitkyina, Myanmar

**Keywords:** psychosocial support, conflict, Myanmar (Burma), internally displaced person (IDP), distance services

## Abstract

**Background:**

In response to the COVID-19 pandemic, the Global Mental Health research group at Johns Hopkins School of Public Health worked with three local partner organizations in Myanmar to develop a psychosocial support (PSS) program that could be delivered by community-based focal points in internally displaced persons camps. This PSS program was designed to be delivered in communities with limited access to regional mental health services due to pandemic travel restrictions. The content of the PSS program was based on materials from an ongoing Common Elements Treatment Approach (CETA) mental health program; CETA counselors based in the three partner organizations were available to provide telephone-based CETA counseling. In April 2020, the partners organizations recruited and trained PSS focal points in 25 IDP camps to establish a multi-tiered system of MHPSS supports.

**Implementation:**

The PSS program including psychoeducation handouts focused on stress and coping during COVID-19 and skills for cognitive restructuring (i.e., changing unhelpful thoughts) in simplified terms, audio recordings of the content of these handouts and referral opportunities for telephone-based services by CETA counselors located outside of the camps. PSS focal points distributed the handouts, had the recordings played *via* radio and loudspeaker, and were available to answer questions and provide access to a PSS program phones to connect with the CETA counselors. After 6 months of implementation, program monitoring logs were reviewed and a cross-sectional evaluation was conducted to assess the PSS program's reach, understanding, and perceived utility.

**Evaluation:**

Forty-one focal points implemented the PSS program in 25 IDP camps in Kachin and northern Shan States. From May to September 2020, the focal points distributed handouts to 5,725 households and reported 679 visits by IDPs, including facilitating 332 calls to a CETA counselor. Data from the program evaluation (*n* = 793 participants) found high levels of handout readership and perceived utility of the information, and good comprehension of the content and application of skills.

**Discussion:**

Findings suggest that provision of a multi-tiered MHPSS program, with PSS focal points providing direct information and linkages to further mental health services *via* telephone, was feasible despite the constraints of the pandemic. Utilizing camp-based focal points was acceptable and successful in delivering basic psychosocial supports to a broad population while serving as points of contact for individuals who wanted and needed telephone-based counseling services.

## Key Findings

- Implementation of a multi-tiered mental health and psychosocial program is feasible, despite the constraints of the COVID-19 pandemic, when higher-tiered level services (e.g., CETA counseling) are already established and accessible.
- Community-based programs utilizing locally recruited focal points was successful in delivering basic psychosocial supports to a broad population while serving as points of contact for individuals who wanted and needed more sustained telephone-based counseling services.
- This program can serve as a model for a multi-tiered MHPSS program.

## Key Implications

- Program managers should consider empowering lay community members to play a role in a multi-tiered MHPSS program, particularly in areas where access to higher level service providers is limited.
- Program managers should consider lay community members as integral components of a responsive system that requires low levels of training and supervision yet allow linkages between community members and MHPSS services.

## Teaser Trailer

During the COVID-19 pandemic, community-based delivery of mental health and psychosocial support information by lay community member focal points proved to be a feasible and acceptable approach for enhancing access to existing remote counseling services.

## Background

COVID-19 significantly impacted provision of mental health and psychosocial support (MHPSS) services. A World Health Organization survey conducted in 2020 found that mental health services were stopped or disrupted in 93% of countries surveyed (1). In addition, the pandemic triggered increased demand for mental health services due to bereavement, fear, and isolation, thus necessitating an increased shift to remote service delivery. In response to the need for digital, telephone, and online MHPSS services, guidelines were developed in real time both at the global level, and also specifically for humanitarian settings (2, 3).

The burden of mental health problems in Myanmar is high across the country with populations in the country's ethnic areas further vulnerable due to ongoing conflict and continued displacement (4, 5). One of the main conflicts in the country is in the northern part of the country in Kachin State (6). In this state, armed conflict between the Kachin Independence Army and government soldiers has been pervasive for decades, most recently reigniting following the collapse of a 17-year ceasefire agreement in 2011. In the 10 years since the end of this ceasefire agreement, fighting escalated and resulted in over 98,000 displaced persons in 171 camps among the over 119,000 people in need of humanitarian assistance in Kachin and northern Shan States (7). With the onset of the COVID-19 pandemic, these camps were put on lockdown, stopping movement of individuals in and out of the camps; over time containment procedures have loosened, but movement is still restricted.

The Global Mental Health group from Johns Hopkins University (JHU) has worked with Myanmar populations since 2008, initially on the Thailand-Myanmar border and then expanding to sites in Myanmar starting in 2015. During this time, JHU has provided training and technical support for five local partner organizations to implement an evidence-based mental health and psychosocial support intervention system, Common Elements Treatment Approach (CETA) (8, 9). Table 1 provides an overview of the key components of the CETA counseling program. Prior to the onset of the COVID-19 pandemic in early 2020, local partner organizations in Kachin and northern Shan States were implementing both CETA through in-person services in camps for internally displaced persons (IDP). Early in the pandemic, training was provided for the CETA providers to pivot to telephone-based counseling.

**Table 1:** Key elements of the Common Elements Treatment Approach (CETA).

**Common Elements Treatment Approach (CETA)**
Psychoeducation
Cognitive coping and restructuring
Safety assessment and planning
Confronting fears and trauma memories
Behavioral activation
Substance abuse intervention
Problem-solving
Anxiety management
Caregiver skills

### Community-Based PSS Program Description

In response to the COVID-19 pandemic, starting in March 2020, JHU worked with the local partners to develop a new community-based PSS program to provide information and immediate supports to the IDPs living in communities that had, due to COVID-19 restrictions, lost in-person access to the available CETA services. Specifically, a community-based program was developed that trained individuals living in the IDP camps, PSS focal points, to deliver written PSS materials related to COVID-19 stress and worries. The materials were developed based on the CETA elements of Psychoeducation, Safety, and Cognitive Coping. Psychoeducation and Safety were included based on the need for foundational information and encouragement for participants and experience from CETA implementing partners that many populations experience challenges to their safety and need this to be confronted early on and during every contact. Cognitive Coping was selected based on evidence of its effectiveness and initial feedback from local CETA implementing partners that it is the most appreciated and most used element of CETA by both providers and clients (10).

The specific content of the PSS materials was developed based on feedback from local partners who had informally collected information from IDP beneficiaries on the stresses they were facing at the beginning of the pandemic. JHU and local partners co-developed the handout information and discussed feasible options for dissemination and linking communities to the CETA counselors. In addition to being trained in the written materials, the focal points were trained to ask simple PSS questions and provide access to a project phone for IDPs who wanted to talk with one of the CETA counselors associated with the partner organizations. These CETA counselors were then able to conduct a structured, validated mental health assessment, and determine if the CETA intervention was appropriate. These counselors were located geographically close to the IDP camps (Laiza and Myikyina areas in Kachin State), had existing familiarity with the IDP camp communities based on previous service provision of CETA, and could provide services in the local languages of the IDP populations (11). The PSS program also included audio recordings of the PSS materials that were regularly broadcast on camp loudspeakers. In April 2020, the three local partner organizations, Kachin Baptist Convention (KBC), Kachin Development Group (KDG), and Substance Abuse Research Association (SARA), began to recruit and train PSS focal points in IDP camps.

## Focal Point Program Implementation

### Intervention Design

Individuals trained in the PSS focal point program were recruited by the three local partner organizations from among individual members living in the IDP camps. The selection criteria were: (1) ability to read and write the local language, (2) available time and interest to support their community during COVID-19, and (3) ability to use social media. PSS focal point backgrounds ranged from having experience with public health program community mobilization to being a school teacher or other community volunteer. The PSS focal points were not considered to be mental health service providers but, instead, were there to disseminate PSS materials in a variety of formats and to act as a conduit for the IDPs to access the CETA mental health counseling services by phone.

The focal points received remote training due to COVID-related travel restrictions. Remote training (by KM) involved sending the PSS handouts and a procedures manual to each of the focal points (both electronic and print versions delivered by the partner organization to the camps) and having the review the materials on their own. This was followed by a training call (small groups and individual calls arranged by organization and availability of the focal points) to review the materials, conduct role plays and review program implementation processes.

The written PSS materials were developed specifically for this program. The first was designed to provide basic psychoeducation, stress and coping management; it focused on mental health information and outlined, in detail, actions for dealing with stress and coping during COVID-19. The information in this handout was in line with World Health Organization (WHO) general information on mental health during the pandemic (12), but provided more details on how and why to respond to mental health problems resulting from the pandemic—both the disease and the control measures. The second handout, based on the CETA Cognitive Coping element, provided information on “changing unhelpful thoughts,” which explained the process of cognitive coping in simplified terms. This handout was based on the CETA intervention component “thinking in a different way” that was adapted for use with these IDP communities to introduce the relationship between thoughts, feelings and behaviors and provide skills and practice for changing one's thoughts about a situation that, itself, cannot be changed. In partnership with The Refugee Response, we also produced a 4-part series of videos and audio recordings, which were made available to the PSS focal points to share with IDPs from their own phones and the audio recordings were shared *via* camp loudspeakers (9).

For program implementation, the PSS focal points received a program kit that included multiple copies of each handout to be given to the IDPs and electronic copies of the 4-part video series and accompanying audio, and a project phone and phone credit to allow IDPs to call the CETA counselors. PSS focal points were trained to make private space in front of their home available for IDPs to choose to use when calling the CETA counselors. Face masks, hand sanitizer, and guidance on how to clean the phones before and after sharing with others were also provided for COVID-19 transmission prevention. The PSS focal points received monthly compensation based on the internal pay scales of their respective organization and in-line with compensation provided to similar positions.

In each camp, the PSS focal points were given a “catchment” area to provide this program. The goal was for the PSS focal points to distribute the handouts to every household in their catchment area; first they distributed the “managing stress and coping” handout and then after 4 weeks, they returned to each household and distributed the “changing unhelpful thoughts” handout. The reason for the staggered distribution was to support multiple visits by the focal points to each household and ongoing presence in the area for individuals who had questions or wanted more support. The PSS focal points also posted the handouts in public areas, shared the videos on social media, and broadcast the audio files on local radio stations and loudspeakers.

During program implementation, the PSS focal points received weekly supervision *via* telephone from a supervisor based with each of the partner organizations. During these calls, the supervisor reviewed how many households had been visited and handouts distributed, how many IDPs had talked with the focal point to get more information MHPSS and available supports, and how many IDPs had used the focal point's phone to call a CETA counselor. In addition, the supervisor solicited information from each focal point about any adjustments that needed to be made to the handouts or process for the program's implementation.

### Setting

The PSS focal point program was conducted in 25 IDP camps in Kachin and northern Shan States. Locations of the camps varied from within city and town centers to remote places in both government and non-government-controlled areas. Camp sizes ranged from 10 to 1,655 households. The total population in the 25 sites was ~34,323 (45.34% under 18 years of age; 54.66% 18 years of age or older; females 51.11%).

### Program Evaluation

A program evaluation was conducted after the PSS focal point program had been implemented in the 25 camps for 6 months; the evaluation focused on program reach (i.e., how many households had received the PSS materials) and utility (i.e., understanding and self-reported use) of the PSS materials. A cross-sectional study design was used to collect qualitative and quantitative information using an online-adaptive, phone-based data collection application (www.kobotoolbox.org) that changed based on the participant's answers. Questions gathered information on receipt of the handouts (yes/no), hearing the audio files (yes/no), use of the skills presented in the handouts, perceived utility of the content in the handouts, and comprehension of the PSS content. Use of skills was assessed by asking about frequency of use for each skill (daily or almost daily, a few times a week or sometimes, never). Perceived utility for each handout was assessed on a 3-point scale (very useful, somewhat useful, not useful). Separate sets of comprehension questions were developed for each handout. Comprehension of the “stress and coping” handout content was assessed by (1) number of signs of stress correctly recalled; (2) correctly identifying steps for supporting themselves or others that were described in the handout (the questionnaire including six correct and two incorrect steps) and (3) correctly identifying a sign that someone is experiencing stress from three scenarios included in the questionnaire. Comprehension of the “changing unhelpful thoughts” handout content was assessed by asking the respondent to create a more helpful thought in response to two example scenarios. To reduce participant burden, each evaluation participant was asked to report on their experience with only one of the handouts.

Thirty-two data collectors with no prior role in the focal point program were trained in basic research ethics, the study questionnaires, and use of the data collection phone application. Training of data collectors was conducted remotely by study staff (KM) in Myanmar language. Data collectors were located in the IDP camps and were able to move freely within the camps to collect data during the program evaluation period. During data collection, study staff maintained daily contact with data collectors to answer questions and confirm the number of questionnaires completed.

Data collectors were given a random start and skip number generated based on the number of houses in their assigned area. Data collectors used this random number to approach houses for participation in the study, alternating between questionnaires for every other house, until they completed their assigned number of interviews for both.

Using standard sample size calculations for population estimates for our knowledge, attitudes and practice questions we calculated needing *n* = 400 completed surveys in order to get 95% confidence with 5% error. Therefore, our aim was to survey 800 adults in order to get estimates for the two different questionnaires.

A total of 793 participants participated in the evaluation; 400 responded to the questionnaire on their experience with the “stress and coping” handout and 393 for the questionnaire on the “changing unhelpful thoughts” handout. Oral consent was obtained from all participants prior to their participation. Data collection took place from October 5, 2020 to October 31, 2020. This program evaluation was approved by the JHU IRB (IRB# 13256).

Data from this data collection effort were analyzed together with process indicators collected as part of regular program monitoring. The PSS focal points kept program logs with information on the number of households visited each day to distribute handouts, the number of IDPs who approached the focal points for further information and support, and the number of times an IDP used the focal point's program phone to talk with a CETA counselor. These logs were reviewed with supervisors each week who entered data into the program monitoring data system.

## Focal Point Program Process Indicators and Evaluation Findings

### Process Indicators

Forty-one focal points (10 male, 31 female), ranging in age from 19 to 42 years old, implemented this PSS program in the 25 IDP camps. From May to September 2020, the PSS focal points distributed handouts to 5,725 households. The PSS focal points were active for an average of 15 non-consecutive weeks in this 5-month period.

Analysis of focal point logs from weekly supervision data identified 679 visits by IDPs to the PSS focal points. During these visits, the PSS focal points reported reviewing the handouts with 521 (76.7%) people. In addition, 332 (48.9%) of the 679 visits resulted in a phone call to a CETA counselor.

### Evaluation Findings

Evaluation respondents ranged in age from 18 to 85 years (mean age 42.5 years). For both questionnaires, there were more female than male respondents (83.75% female respondents for “stress and coping” handout and 84.48% for the “changing unhelpful thoughts” handout).

### Program Reach

Over 93% of respondents for both questionnaires reported receiving the handout with over 85% of these respondents reporting having read the handout (85.83% for the “stress and coping” handout and 86.38% the “changing unhelpful thoughts” handout).

### Use of Information in Handouts

Frequency of using the skills and information in the “stress and coping” handout was reported by 29.28% of respondents to be daily or almost daily, 65.11% reported using the skills a few times a week or sometimes (Table 2). For the “changing unhelpful thoughts” handout, 29.97% of respondents reported using the skills daily or almost daily and 65.62% reported using them a few times a week or sometimes (Table 3).

**Table 2:** Frequency of using the skills or advice in the “managing stress and coping” handout.

**Organization**	**Never**	**A few times a week/ sometimes**	**Daily or almost daily**	**Don't know/no response**
KBC (*n* = 122)	2 (1.64%)	80 (65.57%)	36 (29.51%)	4 (3.28%)
KDG (*n* = 104)	3 (2.88%)	66 (63.46%)	34 (32.69%)	1 (0.96%)
SARA (*n* = 95)	8 (8.42%)	63 (66.32%)	24 (25.26%)	0 (0.00%)
Total (*n* = 321)	13 (4.05%)	209 (65.11%)	94 (29.28%)	5 (1.56%)

**Table 3:** Frequency of using the skills or advice in the “changing unhelpful thoughts” handout.

**Organization**	**Never**	**A few times a week/ sometimes**	**Daily or almost daily**	**Don't know/no response**
KBC (*n* = 121)	2 (1.65%)	80 (66.12%)	37 (30.58%)	2 (1.65%)
KDG (*n* = 108)	1 (0.93%)	75 (69.44%)	29 (26.85%)	2 (1.85%)
SARA (*n* = 90)	5 (5.56%)	53 (58.89%)	29 (32.22%)	2 (2.22%)
Total (*n* = 317)	8 (2.52%)	208 (65.62%)	295 (29.97%)	5 (1.58%)

### Stress and Coping Knowledge

Of the respondents who reported reading the managing “stress and coping” handout (*n* = 321), 42.68% (*n* = 137) correctly recalled 3 or more signs of stress mentioned in the handout. An additional 44.24% (*n* = 142) correctly recalled 1 or 2 signs of stress. For the identifying correct steps for supporting self and others question, 64.80% (*n* = 208) of respondents correctly recalled one or more of the steps from the handout, with 33.64% (*n* = 108) only recalling steps not listed in the handout and 1.56% (*n* = 5) respondents being unable to recall any steps. For the question about identifying someone who is experiencing stress, 72.59% (*n* = 233) of the respondents were able to identify the correct scenario; 25.86% (*n* = 83) selected an incorrect scenario and 1.56% (*n* = 5) did not answer.

### Changing Unhelpful Thoughts Knowledge

In the knowledge assessment, of the 317 respondents who reported reading the managing “changing unhelpful thoughts” handout, 45.11% (*n* = 143) were able to respond with a more helpful thought to the first scenario, while 47.32% (*n* = 150) provided answers that were not considered to be more helpful or were unclear and 7.57% (*n* = 24) were unable to answer. For the second scenario, 63.09% (*n* = 200) were able to create a more helpful thought, while 29.34% (*n* = 93) responded with incorrect or unclear answers and 7.54% (*n* = 24) were unable to provide an answer.

### Usefulness

The information in the “stress and coping” handout was reported to be very useful by 72.27% (*n* = 232), somewhat useful by 27.10% (*n* = 87), and not at all useful by 0.31% (*n* = 1) of respondents who read the handout. This is similar to responses regarding the information in “changing unhelpful thoughts” handout, with 79.18% (*n* = 251) of the respondents who read the handout reporting that the information was very useful and 21.14% (*n* = 67) reporting it to be somewhat useful.

All respondents, including those who had and had not read the print material, were asked to report on whether they heard the information over loudspeaker and/or radio and, if so, how they rated the usefulness of the information. The majority of respondents reported hearing the information over loudspeaker or radio (94.25% for Questionnaire 1 and 96.4% for Questionnaire 2).

The managing stress and coping information played on loudspeakers and/or the radio was reported to be “very useful” by 79.05% (*n* = 298), “somewhat useful” by 20.69% (*n* = 78), and “not at all useful” by 0.27% (*n* = 1) of respondents out of the 379 respondents who reported hearing the audio information. Again, this is very similar to responses regarding the changing unhelpful thoughts handout where 80.74% (*n* = 306) reported the audio information to be very useful and 19.26% reported it to be somewhat useful (*n* = 73), among those who reported hearing the information over loudspeaker and/or radio.

## Discussion

**Figure 1:**
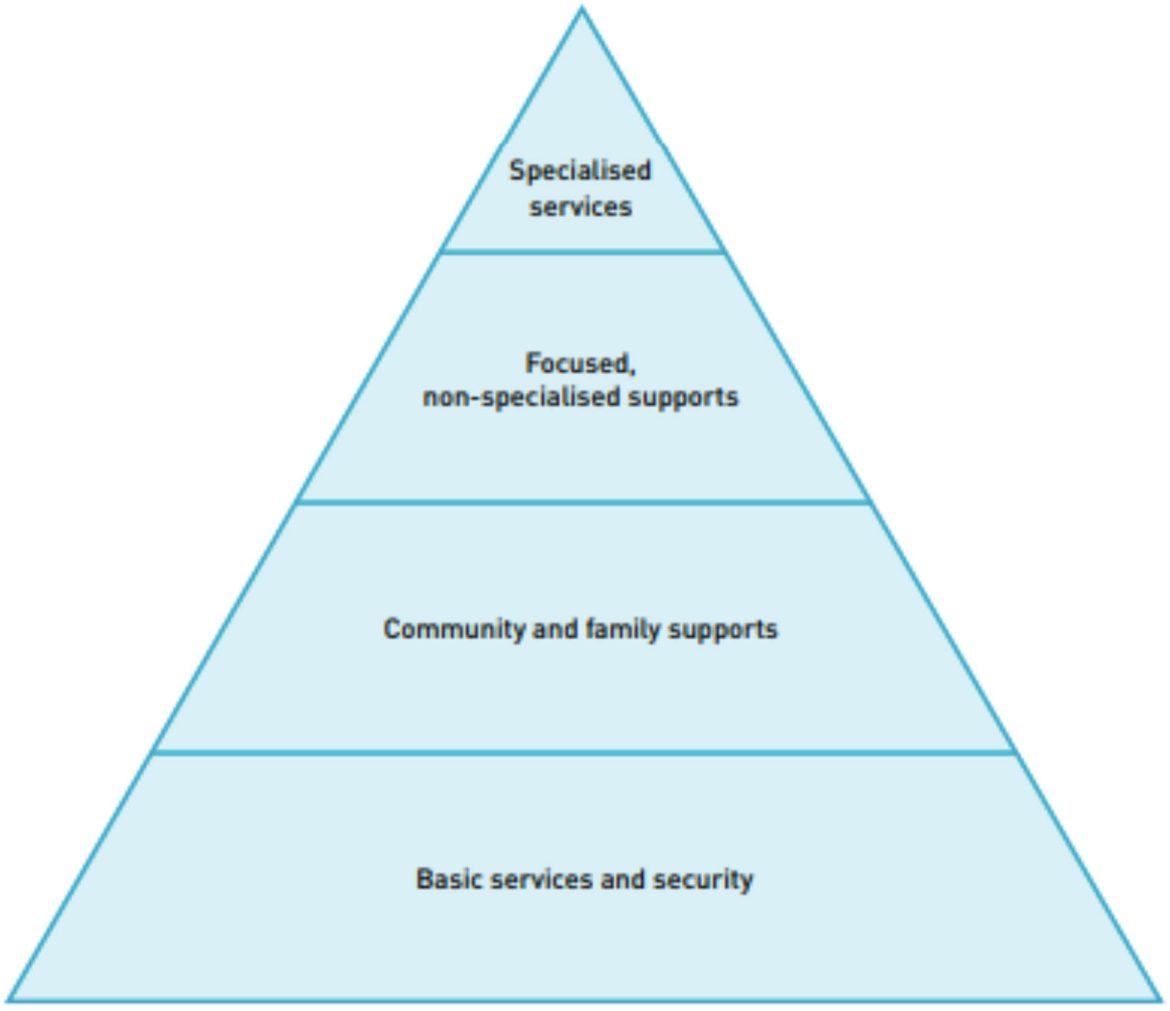
Inter-Agency Standing Committee pyramid of mental health and psychosocial support interventions.

Building off an existing, well-established mental health program with local program implementers, JHU and local partners were able to quickly adapt and implement this PSS focal point program in response to COVID-19. Although approaching MHPSS and PSS service implementation with a community-based focus is commonly found (13, 14), few programs are designed to provide a direct link between the community-based psychosocial programming and more extensive mental health service provision. This gap in multi-tiered service systems exists despite the IASC guidance on MHPSS in emergency settings acknowledging that people in emergencies require different kinds of supports and organizing supports in a layered system of complementary supports is ideal to meeting the different types and severity of needs (Figure 1) (15). This PSS focal point program was specifically designed to be part of a broader MHPSS system of care, providing psychoeducation and basic supports to a broad population and supporting referral and linkage to more specialized services through the direct link between the focal points and the existing CETA counselors. In addition to dissemination of PSS information, the PSS focal points were trained and supervised to both encourage individuals to call a CETA counselor if they wanted more support and to actually facilitate the call using the PSS program phone. As residents in their respective IDP camps, these PSS focal points provided an essential “on the ground” presence during the time of COVID-19.

The broad reach of the program handouts demonstrates that it was both feasible and acceptable to use the PSS focal points to deliver these messages to camp households. In addition, the high percentages of people who reported reading the handouts among the people approached to complete the questionnaires shows that, at least among the people who completed the questionnaires, there was enough local literacy to read and understand the handouts. In addition, everyone who completed the questionnaires said that they heard the PSS content on the loudspeaker and/or radio which provides an additional feasible way to share information in these camps. For both the print and audio information, a high percentage of respondents reported that they found the information useful.

For the assessment of comprehension, respondents' answers to the questions that prompted them to recall and apply specific skills presented in the PSS materials show that there is room for improvement in making sure that community members understand and can use the skills in their daily lives.

### Research Challenges and Research Strategies

While challenges in this humanitarian setting are to be expected, implementation of both the PSS focal point program and the program evaluation data collection efforts faced additional challenges specific to the COVID-19 situation. These challenges were rooted in the difficulty posed by travel restrictions.

Trainings for the PSS focal points and data collectors were conducted remotely due to travel restrictions imposed by the national, state and camp authorities, as well as safety precautions of the research team and its implementing partners. These remote trainings prompted several issues that may have been identified and mitigated more succinctly with in person trainings.

In-person trainings of data collectors typically allows for working closely with trainees to ensure their understanding of the materials and provides opportunities to practice using the data collection tools. The inability to meet in person meant that organizations and data collectors had to receive remote support for loading the data collection platform onto their mobile devices, thus limiting live practice time to troubleshoot devices and ensure the application was properly installed, ready for use and the users understood how to navigate the application. Improper setup of the application caused issues with devices not being connected to the server and therefore, not uploading data to the cloud database.

For the PSS focal points, the remote training meant that, similar to the data collectors, there was a lack of in-person one-on-one observation of role plays which could have better prepared PSS focal points for talking with community members.

To overcome the challenge of remote training for both PSS focal points and data collectors, individual calls were done to provide support and supervision.

Findings from this research support the recognized need for community-based PSS programming that is linked to multi-level services. At a policy level, these findings support efforts to link community-based initiatives with multi-level services. MHPSS needs to have referral between tiers and this can be achieved by shifting policy from a focus on individual levels to one that seeks to meet the goal of multi-level, linked services.

## Conclusions

The goal of this PSS program was to provide psychosocial support to a vulnerable IDP population in the midst of the COVID-19 pandemic and to maintain the connection between the IDP population and the CETA program that was being implemented prior to the lock downs and closures. This program evaluation was implemented to understand the extent to which community members would accept, understand, and apply the information and skills in the PSS handouts and to evaluate the use of the PSS focal points as a point of contact for asking PSS questions and using the PSS program phones to connect with the CETA counselors. Findings suggest that the PSS focal points were able to distribute the PSS materials to a broad set of IDP households and that IDPs not only recognized the PSS focal points as points of contact for mental health counseling services, but sought them out for additional PSS information as well. Further, the findings point to the PSS materials provided as being useful to community members and accessible for them to understand and practice the skills.

Concurrent with the ongoing restrictions on movement because of COVID-19, in February 2021 Myanmar experienced a military coup with significant repercussions for the safety and security of individuals across the country. The organizations described in this paper have been able to continue this tiered MHPSS system of information dissemination and referral for counseling despite this additional challenge. In response to the need for more broad (i.e., non-COVID specific) PSS information, the PSS handouts have been revised to encompass more examples related to stress and trauma in general and address concerns related to the coup. This PSS focal point program, as part of the larger system of care, serves as a model for responding to MHPSS needs of communities in humanitarian and remote settings. To address sustainability in part, the model is currently being updated to include pre-recorded videos to enhance the remote training of PSS focal points, as well as modules for training organization-level supervisors. Further data will be collected on the implementation of this type of training, as well as ongoing implementation of the focal point system.

Adaptations to the program described in this paper could be made in order to more appropriately match the context of new areas, including a review of individual access to personal phones. Because this study was conducted in IDP camps during COVID-19 lockdowns, more people used the available PSS phones compared to personal phones, however, other settings may find that people have more access to personal phones and choose to use these for calling counselors. In addition, the PSS focal points in this program lived in their respective IDP camps, however, implementing organizations in new settings would need to identify the most appropriate frontline workers. People on the community were selected for this study because of COVID-19 restrictions, however, it may be more appropriate to have focal points who are not neighbors, when possible, which is a decision each organization would need to make on a site-by-site basis.

## Data Availability Statement

The raw data supporting the conclusions of this article will be made available by the authors, without undue reservation.

## Ethics Statement

This study was reviewed by the Johns Hopkins School of Public Health Institutional Review Board (IRB# 13256) and determined to be exempt.

## Author Contributions

CL and MS led the writing of this manuscript with editorial support from JB. KM, GD, and LS contributed specific information for sections related to challenges and overcoming challenges. All authors read and approved the final manuscript and were involved in study planning and implementation and analysis and interpretation of the collected data.

## Funding

This study support for CL, MS, KM, and JB which was provided by the United States Agency for International Development (USAID) under grant number: AID-OAA-LA-15-00003. Funding support for implementation of the focal point system by local partners was provided by Development Alternatives Incorporated. Neither of the funding bodies had a direct role in the design of the program, nor the collection, analysis, and interpretation of data and in writing the manuscript.

## Conflict of Interest

The authors declare that the research was conducted in the absence of any commercial or financial relationships that could be construed as a potential conflict of interest.

## Publisher's Note

All claims expressed in this article are solely those of the authors and do not necessarily represent those of their affiliated organizations, or those of the publisher, the editors and the reviewers. Any product that may be evaluated in this article, or claim that may be made by its manufacturer, is not guaranteed or endorsed by the publisher.

## Abbreviations

CETA: Common Elements Treatment Approach
IDP: Internally Displaced Person
IASC: Inter-Agency Standing Committee
JHU: Johns Hopkins University
KBC: Kachin Baptist Convention
KDG: Kachin Development Group
M&E: Monitoring and Evaluation
MHPSS: Mental Health and Psychosocial Support
PSS: Psychosocial Support
SARA: Substance Abuse Research Association
WHO: World Health Organization.
